# Blood-Based Biomarkers as Predictors of All-Cause Dementia: A 13-Year Follow-Up in the Whitehall II Cohort Study

**DOI:** 10.1212/WNL.0000000000218681

**Published:** 2026-10-23

**Authors:** Séverine Sabia, Julien Dumurgier, Clémence Cavaillès, Ian Meneghel Danilevicz, Aurore Fayosse, Amanda J. Heslegrave, Henrik Zetterberg, Mika Kivimaki, Archana Singh-Manoux

**Affiliations:** 1 Université Paris Cité, INSERM U1153, Epidemiology of Ageing and Neurodegenerative Diseases (EpiAgeing), France;; 2 Faculty of Brain Sciences, University College London, United Kingdom;; 3 Department of Neurodegenerative Disease, University College London, Institute of Neurology, Queen Square, United Kingdom;; 4 Department of Psychiatry and Neurochemistry, Institute of Neuroscience and Physiology, the Sahlgrenska Academy at the University of Gothenburg, Mölndal, Sweden;; 5 Clinical Neurochemistry Laboratory, Sahlgrenska University Hospital, Mölndal, Sweden;; 6 UK Dementia Research Institute at University College London, United Kingdom;; 7 Hong Kong Centre for Neurodegenerative Diseases, InnoHK, China;; 8 Wisconsin Alzheimer's Disease Research Center, University of Wisconsin School of Medicine and Public Health, University of Wisconsin-Madison, WI; and; 9 Centre for Brain Research, Indian Institute of Science, Bangalore, India.

## Abstract

**Background and Objectives:**

Blood-based biomarkers are strongly correlated with amyloid positivity in research settings, but their long-term performance in heterogeneous populations remains unclear. Our aim was to determine their long-term prediction accuracy for incident all-cause dementia on their own and when used alongside known risk factors in community-dwelling older adults.

**Methods:**

This was a retrospective study based on the UK Whitehall II longitudinal, population-based cohort study established in 1985 among civil servants that includes a clinical examination every 4–5 years. Blood samples stored at the 2013-2013 wave were assayed in 2024 for measures of amyloid-beta (Aβ) 42 and 40, glial fibrillary acidic protein (GFAP), neurofilament light (NfL), and phosphorylated tau protein 217 (p-tau217). Incident all-cause dementia, from 2012 to 2013 to 2025, was ascertained from linked medical records, and Cox regression was used to examine associations of biomarkers with dementia, followed by estimation of prediction statistics.

**Results:**

Of the 5,225 (27.2% female) participants (mean age in 2012–2013, 69.4 ± 5.7 years), 516 (30.0% female) had a dementia diagnosis over a 12.2-year median follow-up. Participants with values in the worst quartile of amyloid-beta 42 (Aβ42)/40 ratio (hazard ratio [HR] 1.88; 95% CI 1.57–2.24), p-tau217 (HR 3.74; 95% CI 3.12–4.50), NfL (HR 1.88; 95% CI 1.56–2.28), and GFAP (HR 2.25; 95% CI 1.87–2.72) had higher risk of dementia. The C-index for predicting dementia was highest for p-tau217 (0.693; 95% CI 0.672–0.714), lowest for Aβ42/40 ratio (0.587; 95% CI 0.566–0.608), and was 0.760 (0.738, 0.781) for all biomarkers together. Sensitivity (between 41.7% and 59.5%) and positive predictive value (between 16.4% and 23.5%) of all biomarkers were modest, but specificity (between 76.8% and 78.8%) and negative predictive value (92.3%–94.7%) were good. A second approach to prediction modeling showed a model containing sociodemographic, behavioral, and health-related risk factors to have a C-index of 0.765 (95% CI 0.746–0.785); adding all biomarkers to this model led to a C-index of 0.813 (95% CI 0.795–0.830).

**Discussion:**

The long-term predictive ability of blood-based biomarkers for incident dementia in community-dwelling older adults was modest due to poor sensitivity and similar to that of a model containing known risk factors.

## Introduction

There is growing recognition of the need for early detection of Alzheimer disease (AD) and related dementias.^1^ CSF and neuroimaging measures have been shown to be robust biomarkers for use in trials of disease-modifying drugs^2^ and diagnostic purposes, but cost and invasiveness have restricted their use to specialized research and care settings. In recent years, major advances have been made in identifying blood-based biomarkers of AD; the most promising are measures of amyloid-β (A*β*) species, phosphorylated tau (p-tau), the reactive astrogliosis marker glial fibrillary acidic protein (GFAP), and neurodegeneration marker neurofilament light chain (NfL).^3-6^

A number of recent studies report high accuracy of blood-based biomarkers when tested against amyloid PET^6-10^ or CSF biomarkers,^5,7,11^ with some evidence to suggest that these biomarkers may also be relevant for forms of dementia other than AD.^12^ For p-tau217, these studies report area under the curve (AUC) above 0.90,^5,8,10^ but much of this research remains cross-sectional, where the onset or duration of amyloid positivity is not considered, and the strict inclusion criteria used in these studies raise issues of generalizability. For biomarkers to be useful, they ought to be able to identify people early in the disease process, requiring a longitudinal framework to test their effectiveness. A further consideration is how well these biomarkers work in the general population—in real-life settings among heterogeneous, community-dwelling older adults.

A few longitudinal studies in the past 5 years have used blood-based biomarkers to examine associations with incident all-cause dementia,^13-21^ with follow-up ranging from 3^14^ to 16^21^ years. These studies show low levels of amyloid-beta 42 (Aβ42)^13^ and Aβ42/40 ratio^17^ and high levels of NfL,^13,15,16,18,20,21^ GFAP,^14,15,18,21^ p-tau181,^15,16,21^ and p-tau181/Aβ42 ratio^17^ to be associated with higher risk of all-cause dementia. There remain unanswered questions. Although p-tau217 is thought to be most closely associated with AD brain pathology,^7,9,10,22-25^ only 2^16,21^ of these studies considered it in the biomarker panel and found it to have the strongest association with incident dementia. Prediction accuracy is rarely reported in population-based studies besides notable exceptions. The US-based Atherosclerosis Risk in Communities (ARIC) study (1,525 participants, median follow-up 7.4 years) used measures of amyloid-beta 40 (Aβ40)/42 ratio, NfL, p-tau181, and GFAP and reported an AUC of 0.74 that was no better than the AUC for demographic factors alone at 0.76.^19^ The Swedish National study on Aging and Care in Kungsholmen (SNAC-K) on 2,148 participants (median follow-up 10.2 years) reported AUCs ranging from 0.709 to 0.826 for individual biomarkers for a prediction horizon of 10 years for all-cause dementia,^21^ but the role of sociodemographic and health-related factors was not considered explicitly.

Our aim was to address these limitations by using a panel of biomarkers composed of measures of Aβ40, Aβ42, NfL, p-tau217, and GFAP to examine associations and prediction accuracy in a well-characterized, community-based cohort study with a follow-up for dementia of up to 13 years. We examined the prediction accuracy of biomarkers in 2 ways, first by modeling them on their own to examine their utility when considered on their own and then by examining whether they improved prediction accuracy in models containing sociodemographic and known risk factors to examine their contribution to dementia prediction.

## Methods

### Study Population

The target population for the Whitehall II cohort study was all London-based office staff, aged 35–55 years, working in 20 civil service departments in London at inclusion, between September 10, 1985, and March 29, 1988. The response rate was 73%, and 10,308 (6,895 men and 3,413 women) people were included in the study.^26^ Although most participants were white collar staff, they represented a wide socioeconomic spectrum. The study design consists of a clinical examination that also includes collection of blood samples, a self-reported questionnaire every 4–5 years over the follow-up, along with a linkage to the National Health Service (NHS) electronic health records until 2025.

### Measures

#### Blood-Based Biomarkers

Blood samples were collected at the clinical examination in 2012–2013 by standard venipuncture in EDTA tubes and plasma prepared by centrifugation at 2000 × g for 15 minutes at 4°C within 2 hours after collection. These were then stored in 0.25 mL aliquots at −70°C in the study biobanking facilities and first thawed when transferred to the UK Dementia Research Institute Fluid Biomarker Laboratory in 2024 for assays, undertaken blinded to clinical characteristics of the participants. The Neuro 4-PlexE assay kit (Quanterix, Billerica MA) was used to measure Aβ40, Aβ42, GFAP, and NfL. The ALZpath Simoa pTau-217 v2 Assay (Quanterix HD-X Analyzer platform, MA) was used for the quantitative determination of phosphorylated tau protein 217 (p-tau217).^27^ All coefficients of variation for internal assay controls were below 10%.

#### All-Cause Dementia

Dementia cases were ascertained by linkage to 3 national registers (the national hospital episode statistics [HES] database, the Mental Health Services Data Set, and the mortality register) up March 31, 2025, using each participant's unique NHS identification number. Dementia cases were identified based on ICD-10 codes F00-F03, F05.1, G30, and G31. The NHS provides most of the health care in the United Kingdom, including out-patient and in-patient care. The sensitivity and specificity of dementia diagnoses in the NHS HES data are 78.0% and 92.0%, respectively.^28^ The sensitivity in our study is likely to be higher because we used additional linked data from the Mental Health Services Data Set, a national database which contains information on dementia for persons in contact with mental health services in hospitals, outpatient clinics, and the community.^29^ Date of dementia was set at the first record of dementia diagnosis in any of the 3 databases used for ascertainment.

#### Covariates

Sociodemographic variables included age at baseline (2012–2013), sex (from administrative records), self-reported ethnicity (White, non-White), and education (lower primary school or less, lower secondary school, higher secondary school diploma, university, and higher degree). Behavioral factors measured were smoking status (never, ex-smoker, and current smoker) and physical activity (none, less than recommended, following World Health Organization recommendations of ≥2.5 hours of moderate-to-vigorous physical activity per week).

Health-related covariates included body mass index (BMI) (using measured height and weight and categorized as <25, 25 to 29.9, and ≥30 kg/m^2^), hypertension (ICD-10 I10-I15, systolic/diastolic ≥140/90 mm Hg or use of antihypertensive medication), diabetes (ICD-10 E10-E14, reported doctor-diagnosed diabetes, use of diabetes medication, or fasting glucose ≥7.0 mmol/L), cardiovascular disease (including coronary heart disease (ICD-10 I20-I25) and stroke (ICD-10 I60, I61, I63, I64)), and kidney function using estimated Glomerular Filtration Rate (eGFR) based on the CKD Epidemiology Collaboration equation^30^ and used as eGFR <60 or ≥60.

In the prediction analyses, we also included Mini-Mental Status Examination (MMSE), a global test of cognitive status, and *APOE* genotype (ε4 carriership), determined using a standard PCR assay and the salting-out method.

### Statistical Analysis

Baseline characteristics of the study population were examined as a function of dementia status at the end of the follow-up using chi-square and *t* tests. The mean and SD of biomarkers as a function of baseline characteristics and by dementia and mortality status at the end of follow-up in 5-year age groups were estimated. Histogram density plots were created for each biomarker (Aβ42/40 ratio, p-tau217, NfL, GFAP, measured at baseline) to visualize differences in unstandardized concentration distributions between dementia cases and noncases. All 4 biomarkers were categorized into quartiles and also standardized as Z-scores (mean = 0, SD = 1) after log transformation.

#### Association Between Biomarkers and Risk of Incident Dementia

The shape of the association of biomarkers, using both unstandardized values and Z-scores, with incident dementia was examined using restricted cubic splines regression in analyses adjusted for age, sociodemographic, behavioral, and health-related covariates.

Cause-specific Cox regression, that takes competing risk of mortality into account by censoring at death, was used to examine the association between each plasma biomarker and incident dementia. In additional analyses, the Fine and Gray subdistribution hazard models were used to address competing risk of mortality in an alternative strategy. The timescale was defined as the follow-up duration since baseline (2012–2013), and each participant was followed until dementia diagnosis, end of follow-up, or death, whichever came first.

To examine changes in the hazard ratios, reflecting the association of biomarkers with incident dementia over the follow-up, we also used flexible parametric survival model.^31^ This parametric model and the Schoenfeld's residuals suggested nonproportionality of hazards for some associations leading us to undertake analyses using the total follow-up and also by splitting the follow-up time at 7 years for a short and long follow-up. In these analyses, biomarkers were modeled in quartiles, worst quartile vs others, and also as Z-scores (Aβ42/40 ratio Z-score was inverse coded so that higher values reflected higher risk).

#### Predictive Value of the Biomarkers for Dementia

The predictive performance of the biomarkers was examined in 2 ways:

1. The C-index associated with biomarker positivity (worst quartile) was examined by considering each biomarker separately and then together in 1 model to assess the impact of concomitant pathophysiologic processes. The C-indices were compared using a nonparametric test^32^ with p-tau217 as the reference. In addition, to further assess predictive ability of biomarkers, we used the worst quartile value of the biomarkers to examine sensitivity (true positives), specificity (true negatives), positive predictive value (PPV), and negative predive value (NPV). Because PPV and NPV measures are affected by the prevalence of the outcome, here all-cause dementia, we also simulated these estimates for an incident all-cause dementia rate at 50%, reflecting cross-sectional studies in this domain, for example, amyloid PET positivity at 48.7% in a recent study.^10^

These analyses were repeated by progressively adding covariates in blocks, consisting of age (age and age^2^), sociodemographic factors (sex, ethnicity, and education), behavioral factors (smoking status and physical activity), health-related factors (BMI, kidney function, hypertension, type 2 diabetes, and cardiovascular disease), MMSE, and *APOE* ε4 status. The analyses were undertaken using the total follow-up as well as the follow-up split at 7 years.

In additional exploratory analyses, the predictive ability of biomarkers was examined in analyses stratified by sex and age at baseline.

2. The C-index of a base model containing age, sociodemographic, behavioral, and health-related factors (Model 1) was estimated, and the difference in C-index on addition of biomarkers was calculated. In the next step, the base model also included MMSE (Model 2) and then *APOE* (Model 3).

### Standard Protocol Approvals, Registrations, and Patient Consents

Written informed consent from participants and research ethics approvals were renewed at each contact; the most recent approval was from the University College London Hospital Committee on the Ethics of Human Research, reference number 85/0938.

### Data Availability

Data and study materials that support the analyses presented in this article can be obtained from the corresponding author upon reasonable request, subject to applicable ethical and legal restrictions.

## Results

Of the 10,308 (33% female) participants recruited to the study, a total of 6,308 (29% female) participated in the 2012–2013 wave (eFigure 1), the baseline of this study. Participants excluded from this study were those with dementia diagnosis at baseline (N = 22) or with incomplete data on biomarkers or covariates (N = 1,061), yielding 5,225 (27% female) participants for this study. A total of 516 (30% female) of these participants had a dementia diagnosis by March 2025, over a median follow-up of 12.2 years (IQR 11.6 to 12.6).

Baseline characteristics of the participants by dementia status at the end of the follow-up, presented in Table 1, indicated that those who developed dementia had a lower level of education, were less active, and more likely to have kidney dysfunction, hypertension, diabetes, and cardiovascular disease (all *p* < 0.05). The distribution of biomarkers as a function of covariates, presented in eTable 1, suggested the important impact of older age and kidney dysfunction on biomarker levels. The impact of age was also seen in further assessment of biomarker levels across age groups in analyses stratified first by dementia and then mortality status over the follow-up (eTable 2).

**Table 1:** Participants' Characteristics as a Function of Incident Dementia (Over the Follow-Up From 2012 to 2013 to March 2025)

Characteristics at baseline (2012–2013)	Dementia status at end of follow-up		
	No dementia	Dementia	*p* Value^a^
N	4,709	516	
Age at baseline (y), M (SD)	68.9 (5.6)	74.1 (5.3)	<0.001
Sex			0.132
Male	3,441 (73.1)	361 (70.0)	
Female	1,268 (26.9)	155 (30.0)	
Ethnicity			0.003
White	4,386 (93.1)	462 (89.5)	
Non-White	323 (6.9)	54 (10.5)	
Education			<0.001
Primary school or less	414 (8.8)	79 (15.3)	
Lower secondary school	1,474 (31.3)	188 (36.4)	
Higher secondary school	1,306 (27.7)	112 (21.7)	
University	1,131 (24.0)	100 (19.4)	
Higher degree	384 (8.2)	37 (7.2)	
Smoking status			0.295
Never smoker	2,275 (48.3)	251 (48.6)	
Ex-smoker	2,273 (48.3)	254 (49.2)	
Current smoker	161 (3.4)	11 (2.1)	
Physical activity			<0.001
None	533 (11.3)	89 (17.2)	
Less than recommended	1,658 (35.2)	196 (38.0)	
Recommended	2,518 (53.5)	231 (44.8)	
BMI			0.198
<25 kg/m^2^	1785 (37.9)	211 (40.9)	
25–29.9 kg/m^2^	2018 (42.9)	221 (42.8)	
≥30 kg/m^2^	906 (19.2)	84 (16.3)	
Kidney function (eGFR<60)	509 (10.8)	73 (14.2)	0.022
Hypertension	2,455 (52.1)	328 (63.6)	<0.001
Diabetes	590 (12.5)	88 (17.1)	0.004
Cardiovascular disease	721 (15.3)	119 (23.1)	<0.001
MMSE score, M(SD)	28.4 (1.5)	27.2 (2.5)	<0.001
*APOE* ε4 (1 or 2 alleles)^b^	989 (25.5)	173 (43.1)	<0.001
Plasma biomarkers, M(SD)			
Aβ42/40 ratio (%)	7.6 (2.1)	7.0 (1.9)	<0.001
p-tau217 (pg/mL)	0.31 (0.20)	0.52 (0.35)	<0.001
NfL (pg/mL)	13.2 (12.4)	17.1 (7.9)	<0.001
GFAP (pg/mL)	86.9 (70.5)	122.3 (56.0)	<0.001
Data from the end of the follow-up (March 2025)			
Follow-up period (y), median (IQR)	12.3 (12.0–12.7)	8.1 (4.9–10.3)	<0.001
Age at end of follow-up/dementia diagnosis, M (SD)	80.5 (5.4)	81.6 (5.8)	<0.001
N death during the follow-up period	767 (16.3)	299 (58.0)	<0.001

Abbreviations: Aβ42/40 = the ratio of amyloid-β 42 to amyloid-β 40; BMI = body mass index; eGFR = estimated Glomerular Filtration Rate; GFAP = glial fibrillary acidic protein; IQR = interquartile range; M = mean; MMSE = Mini-Mental State Examination; NfL = neurofilament light chain; p-tau217 = tau phosphorylated at T217.

Data are N (%) unless stated otherwise.

a Differences were tested using a chi-square and Student *t* tests.

b The N cases/total is 401/4,281 due to incomplete *APOE* genotype data.

The density histograms of unstandardized biomarker suggested considerable overlap in values for groups defined by dementia status (at the end of follow-up) for Aβ42/40 ratio but not for p-tau217, NfL, and GFAP (Figure 1, panel A); these differences in distribution persisted when biomarkers were standardized (eFigure 2, panel A).

**Figure 1:**
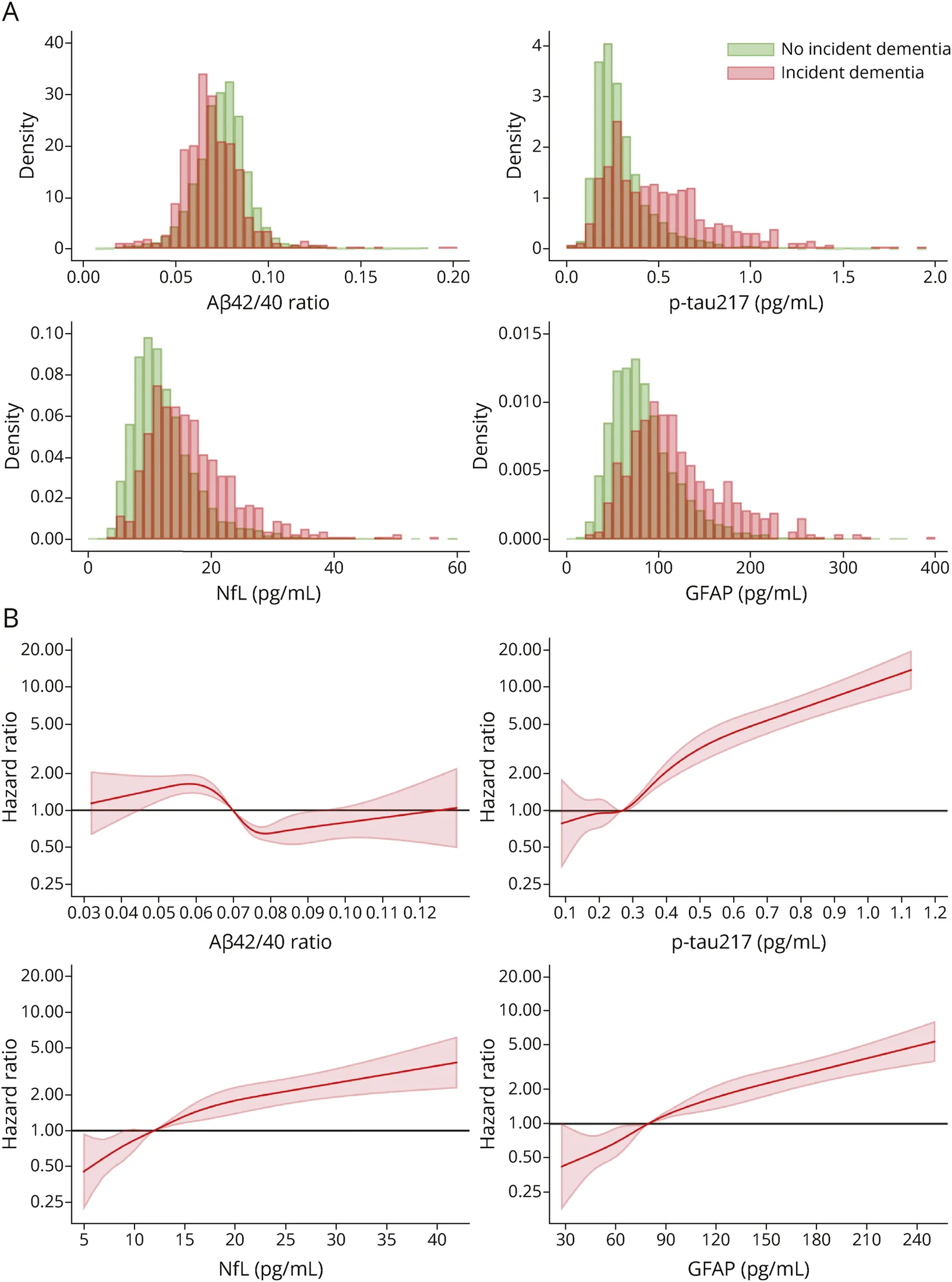
Density Histograms in Panel A and Restricted Cubic Splines for the Association of Biomarkers With Incident Dementia, Follow-Up From 2012 to 2013 to March 2025 (Panel B)^a,b^ Using Unstandardized Values of Biomarkers ^a^ Analyses with the biomarkers entered as restricted cubic splines in Cox regressions adjusted for age, age,^2^ sex, ethnicity, education, smoking status, physical activity, BMI, hypertension, kidney function, type 2 diabetes, and cardiovascular disease. The reference in the spline was the median for each biomarker: 0.07 for Aβ42/40 ratio, 0.27 for p-tau217, 12 for NfL, and 80 for GFAP. ^b^ The *p* values for nonlinearity were Aβ42/40 ratio (*p* < 0.0001), p-tau217 (*p* = 0.008), NfL (*p* = 0.065), and GFAP (*p* = 0.191). Aβ42/40 = the ratio of amyloid-β 42 to amyloid-β 40; BMI = body mass index; GFAP = glial fibrillary acidic protein; NfL = neurofilament light; p-tau217 = phosphorylated tau protein 217.

### Association of Biomarkers With Incident Dementia

NfL and GFAP but not Aβ42/40 ratio or p-tau217 had a fairly linear association with incident dementia in that higher biomarker values were associated with higher risk of incident dementia (Figure 1, panel B) that persisted with standardized values of biomarkers (eFigure 2, panel B). The results of the Cox regression, treating each biomarker in quartiles as well as a Z-score, are presented in Table 2. In analyses adjusted for all covariates (Model 1), participants with values in the worst quartile compared with all others for Aβ42/40 ratio (HR 1.88; 95% CI 1.57–2.24), p-tau217 (HR 3.74; 95% CI 3.12–4.50), NfL (HR 1.88; 95% CI 1.56–2.28), and GFAP (HR 2.25; 95% CI 1.87–2.72) had higher risk of incident dementia. These associations were slightly attenuated when all 4 biomarkers were entered in the Cox regression simultaneously (Model 2, Table 2). Additional analyses using Fine and Gray competing risk analyses yielded results similar to that in the main analyses (eTable 3).

**Table 2:** Association of Blood-Based Biomarkers (Measured in 2012–2013) With Incident Dementia Over the Follow-Up (From 2012 to 2013 to March 2025)

		Model 1^a^		Model 2^b^	
	N cases/N total (%)	HR (95% CI)	*p* Value	HR (95% CI)	*p* Value
Aβ42/40 ratio (%)					
Q1 (0.64–6.57)	215/1,307 (16.5)	2.12 (1.64–2.74)	<0.001	1.47 (1.13–1.91)	0.001
Q2 (6.58–7.47)	128/1,306 (9.8)	1.34 (1.01–1.77)	0.042	1.10 (0.83–1.46)	0.304
Q3 (7.48–8.29)	89/1,306 (6.8)	1.01 (0.75–1.37)	0.933	1.00 (0.74–1.35)	0.840
Q4 (8.30–55.4)	84/1,306 (6.4)	Ref		Ref	
Q1 vs others	215/1,307 (16.5)	1.88 (1.57–2.24)	<0.001	1.41 (1.18–1.70)	0.001
Z-score^c^	516/5,225 (9.9)	1.20 (1.12–1.28)	<0.001	1.11 (1.02–1.20)	0.012
p-tau217 (pg/mL)					
Q1 (0.02–0.19)	54/1,307 (4.1)	Ref		Ref	
Q2 (0.20–0.26)	65/1,306 (5.0)	1.05 (0.73–1.50)	0.808	0.99 (0.69–1.43)	0.999
Q1 (0.27–0.37)	90/1,306 (6.9)	1.31 (0.93–1.85)	0.120	1.14 (0.81–1.60)	0.493
Q4 (0.38–6.30)	307/1,306 (23.5)	4.26 (3.16–5.75)	<0.001	3.03 (2.22–4.13)	<0.001
Q4 vs others	307/1,306 (23.5)	3.74 (3.12–4.50)	<0.001	3.01 (2.48–3.66)	<0.001
Z-score^c^	516/5,225 (9.9)	1.97 (1.82–2.14)	<0.001	1.75 (1.60–1.92)	<0.001
NfL (pg/mL)					
Q1 (1.26–8.99)	42/1,307 (3.2)	Ref		Ref	
Q2 (9.00–11.73)	78/1,306 (6.0)	1.44 (0.98–2.10)	0.062	1.21 (0.82–1.78)	0.333
Q3 (11.74–15.57)	143/1,306 (11.0)	2.03 (1.42–2.90)	<0.001	1.50 (1.03–2.17)	0.038
Q4 (15.57–496.47)	253/1,306 (19.4)	3.11 (2.18–4.42)	<0.001	1.77 (1.21–2.59)	0.009
Q4 vs others	235/1,306 (19.4)	1.88 (1.56–2.28)	<0.001	1.33 (1.09–1.63)	0.006
Z-score^c^	516/5,225 (9.9)	1.48 (1.34–1.63)	<0.001	1.08 (0.97–1.22)	0.171
GFAP (pg/mL)					
Q1 (8.08–59.78)	47/1,307 (3.6)	Ref		Ref	
Q2 (59.79–80.29)	69/1,306 (5.3)	1.16 (0.80–1.69)	0.440	0.98 (0.67–1.44)	0.890
Q3 (80.30–107.75)	129/1,306 (9.9)	1.83 (1.30–2.58)	0.001	1.30 (0.91–1.85)	0.169
Q4 (107.76–3,875.21)	271/1,306 (20.8)	3.21 (2.30–4.49)	<0.001	1.72 (1.20–2.46)	0.005
Q4 vs others	271/1,306 (20.8)	2.25 (1.87–2.72)	<0.001	1.54 (1.26–1.88)	<0.001
Z-score^c^	516/5,225 (9.9)	1.72 (1.56–1.88)	<0.001	1.34 (1.20–1.49)	<0.001

Abbreviations: Aβ42/40 = the ratio of amyloid-β 42 to amyloid-β 40; BMI = body mass index; GFAP = glial fibrillary acidic protein; HR = hazard ratio; NfL = neurofilament light; p-tau217 = phosphorylated tau protein 217.

a Model 1: adjusted for age, age,^2^ sex, ethnicity, education, smoking status, physical activity, BMI, hypertension, kidney function, type 2 diabetes, and cardiovascular disease.

b Model 2: Model 1 + all biomarkers entered in the model simultaneously.

c Log-transformed variables were Z-scored (mean = 0, SD = 1), inverse coding for Aβ42/40 ratio.

There was some evidence of change in the hazard ratios over the follow-up (eFigure 3), confirmed in violation of the proportional hazards assumption, particularly for p-tau217 and NfL. Cox regression with the follow-up for incident dementia split at 7 years, presented in eTable 4, suggested stronger associations in the short-term compared with the long-term follow-up. This pattern persisted when biomarkers were entered in the model simultaneously (eTable 5), with stronger associations of GFAP with incident dementia in the longer follow-up.

### Predictive Performance of Biomarkers for Incident Dementia

The predictive ability of biomarkers is shown in Figure 2 for the total follow-up and the follow-up split at 7 years. Over the total follow-up, the C-index without other covariates in the model (unadjusted model) was highest for p-tau217 (0.693; 95% CI 0.672–0.714), lowest for Aβ42/40 ratio (0.587; 95% CI 0.566–0.608), and was 0.760 (0.738, 0.781) when all biomarkers were included together in the prediction model. Additional analyses, presented in Table 3, suggested modest sensitivity and specificity for all biomarkers, with the highest values at 59.5% and 78.8% for p-tau217, respectively. The PPV for all biomarkers was low, ranging from 16.4% to 23.5%. By contrast, the NPVs were good for all biomarkers, ranging from 92.3% (Aβ42/40 ratio) to 94.7% (p-tau217). When rate of all-cause dementia over the follow-up was set at 50%, the PPVs were higher but NPVs were lower than that obtained with observed rate of all-cause dementia (9.9%).

**Figure 2:**
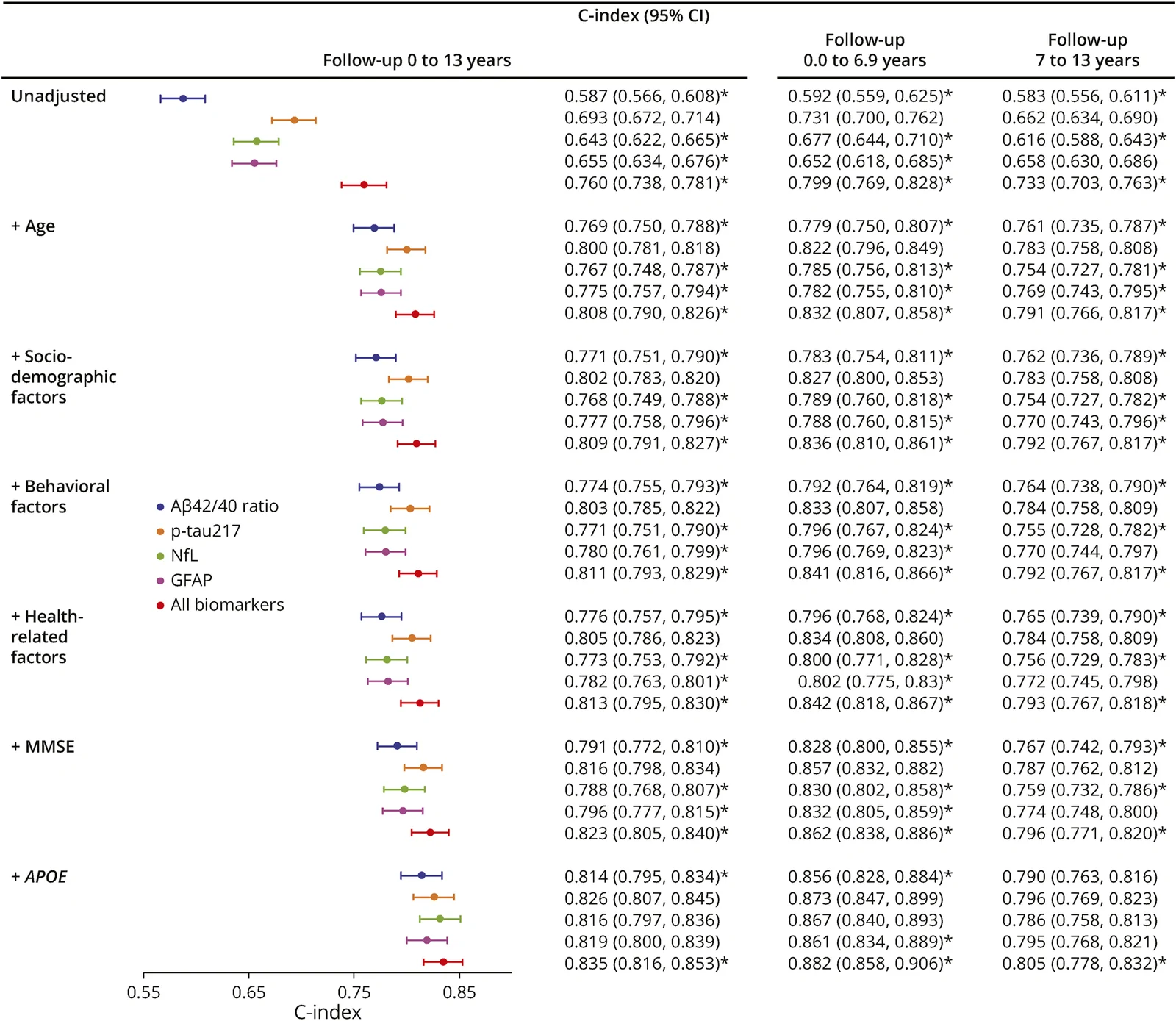
C-Index of Biomarkers (Measured in 2012–2013) for Incident Dementia (Follow-Up From 2012 to 2013 to March 2025) in Unadjusted Models and Then With Progressive Inclusion of Covariates in the Model^a,b,*^ ^a^ The analyses are based on 516 incident cases of dementia in 5,225 participants over the 13-year follow-up; the N cases/N Total for the 0–6.9 years follow-up was 210/5,225, and for the 7–13 years follow-up, it was 306/4,677. ^b^ Analyses adjusted in blocks for age (age, age^2^), sociodemographic factors (sex, ethnicity, and education), behavioral factors (smoking status, physical activity), cardiometabolic factors (BMI, hypertension, kidney function, type 2 diabetes, and cardiovascular disease), MMSE, and *APOE* genotype. (*) Denotes differences at *p* 0.05 with p-tau217 as the reference. Aβ42/40 = the ratio of amyloid-β 42 to amyloid-β 40; BMI = body mass index; GFAP = glial fibrillary acidic protein; MMSE = Mini-Mental Status Examination; NfL = neurofilament light; p-tau217 = phosphorylated tau protein 217.

**Table 3:** Sensitivity and Specificity of Biomarkers With Observed Incident Dementia Rate and Estimated With a Scenario of Incident All-Cause Dementia at 50%

		Sensitivity (%)	Specificity (%)	Observed incident dementia rate (9.9%)		Scenario of incident dementia rate at 50%	
	N cases/total			PPV (%)	NPV (%)	PPV (%)	NPV (%)
Aβ42/40 ratio							
All others	301/3,918						
Worst quartile	215/1,307	41.7	76.8	16.4	92.3	64.2	56.8
p-tau217 (pg/mL)							
All others	209/3,919						
Worst quartile	307/1,306	59.5	78.8	23.5	94.7	73.7	66.0
NfL (pg/mL)							
All others	263/3,919						
Worst quartile	253/1,306	49.0	77.6	19.4	93.3	68.7	60.4
GFAP (pg/mL)							
All others	245/3,919						
Worst quartile	271/1,306	52.5	78.0	20.8	93.7	70.5	62.2
One biomarker in worst quartile							
All others	74/2,255						
Worst quartile	442/2,970	85.7	46.3	14.9	96.7	61.5	76.4
Two biomarkers in worst quartile							
All others	193/3,777						
Worst quartile	323/1,448	62.6	76.1	22.3	94.9	72.4	67.0
Three biomarkers in worst quartile							
All others	307/4,586						
Worst quartile	209/639	40.5	90.9	32.7	93.3	81.6	60.4
All 4 biomarkers in worst quartile							
All others	444/5,057						
Worst quartile	72/168	14.0	98.0	42.9	91.2	87.3	53.2

Abbreviations: Aβ42/40 = the ratio of amyloid-β 42 to amyloid-β 40; GFAP = glial fibrillary acidic protein; NfL = neurofilament light; NPV = negative predictive value; PPV = positive predictive value; p-tau217 = phosphorylated tau protein 217.

Sensitivity = True positive / (True positive + False negative).

Specificity = True negative / (True negative + False positive).

PPV = (Sensitivity × prevalence) / (Sensitivity × prevalence + [1 - Specificity] × [1-prevalence]).

NPV = (Specificity × [1—prevalence] / (Specificity × [1—prevalence] + [1—Sensitivity] × prevalence).

In subsequent analyses, covariates were added successively to the prediction model, the age block led to the largest increases in C-index, an increment of 0.182 for Aβ42/40 ratio to 0.107 for p-tau 217 (Figure 2). Subsequent addition of covariates led to small changes in the C-index, for example, from 0.010 to 0.028 for *APOE* genotype. The C-index for p-tau217 was greater than other biomarkers when they were considered individually, except the models that also contained *APOE* genotype. The prediction model with all biomarkers considered together had higher C-index than p-tau217 alone, irrespective of the covariates included in the model. The pattern of results was similar in analyses split by follow-up period, with some evidence of better prediction at the shorter follow-up.

Additional exploratory analyses stratified by sex with 361 dementia cases in men (9.5%) and 155 (10.9%) showed similar patterns, in that p-tau217 had the highest predictive performance (eTable 6). Splitting age at biomarker measurement (younger than 75 years, 75 years or older) suggested somewhat better predictive ability of biomarkers in the younger age group (eTable 6).

In subsequent analyses, we examined whether biomarkers added predictive value to a model containing sociodemographic, behavioral, and health-related risk factors (Model 1, Table 4) that had a C-index of 0.765 (95% CI 0.746–0.785). The estimates for difference in C-index showed small improvements in prediction with the addition of each biomarker individually and then all together (C-index 0.813; 95% CI 0.795–0.830). A similar pattern was observed when MMSE (Model 2) and also *APOE* genotype (Model 3) were included in the prediction model. This pattern of results was similar in analyses stratified by length of follow-up.

**Table 4:** Predictive Performance of Blood-Based Biomarkers (Using Worst Quartile) for Incident Dementia Over a 13-Year Follow-Up

	Follow-up 0–13 y		Follow-up 0–6.9 y		Follow-up 7–13 y	
	C-index (95% CI)	Difference in C-index (95% CI)	C-index (95% CI)	Difference in C-index (95% CI)	C-index (95% CI)	Difference in C-index (95% CI)
Model 1 (M1): sociodemographic, behavioral, health-related factors^a^	0.765 (0.746 to 0.785)	Ref	0.787 (0.758 to 0.816)	Ref	0.753 (0.726 to 0.780)	Ref
M1 + Aβ42/40 ratio	0.776 (0.757 to 0.795)	0.011 (0.005 to 0.017)^a^	0.796 (0.768 to 0.824)	0.009 (0.001 to 0.018)^a^	0.765 (0.739 to 0.790)	0.012 (0.003 to 0.020)^a^
M1 + p-tau217	0.805 (0.786 to 0.823)	0.039 (0.028 to 0.051)^a^	0.834 (0.808 to 0.861)	0.048 (0.029 to 0.066)^a^	0.784 (0.758 to 0.809)	0.031 (0.016 to 0.046)^a^
M1 + NfL	0.773 (0.753 to 0.792)	0.007 (0.002 to 0.013)^a^	0.800 (0.771 to 0.828)	0.013 (0.003 to 0.023)^a^	0.756 (0.729 to 0.783)	0.003 (−0.002 to 0.009)
M1 + GFAP	0.782 (0.763 to 0.801)	0.017 (0.009 to 0.024)^a^	0.802 (0.775 to 0.830)	0.015 (0.005 to 0.025)^a^	0.772 (0.745 to 0.798)	0.019 (0.008 to 0.030)^a^
M1 + Aβ42/40, p-tau217, NfL, GFAP	0.813 (0.795 to 0.830)	0.047 (0.034 to 0.060)^a^	0.844 (0.819 to 0.868)	0.057 (0.038 to 0.076)^a^	0.793 (0.767 to 0.818)	0.040 (0.023 to 0.058)^a^
Model 2: Model 1+MMSE	0.782 (0.763 to 0.802)	Ref	0.823 (0.795 to 0.852)	Ref	0.756 (0.729 to 0.782)	Ref
M2 + AB42/40 ratio	0.791 (0.772 to 0.81)	0.009 (0.003 to 0.014)^a^	0.828 (0.800 to 0.855)	0.004 (−0.002 to 0.01)	0.767 (0.742 to 0.793)	0.012 (0.003 to 0.020)^a^
M2 + p-tau217	0.816 (0.798 to 0.834)	0.033 (0.022 to 0.044)^a^	0.857 (0.832 to 0.882)	0.033 (0.018 to 0.049)^a^	0.787 (0.762 to 0.812)	0.031 (0.016 to 0.046)^a^
M2 + NfL	0.788 (0.768 to 0.807)	0.005 (0.000 to 0.010)^a^	0.830 (0.802 to 0.858)	0.007 (−0.002 to 0.015)	0.759 (0.732 to 0.786)	0.003 (−0.002 to 0.009)
M2 + GFAP	0.796 (0.777 to 0.815)	0.014 (0.007 to 0.021)^a^	0.832 (0.805 to 0.859)	0.008 (0.001 to 0.016)^a^	0.774 (0.748 to 0.800)	0.019 (0.008 to 0.030)^a^
M2 + Aβ42/40, p-tau217, NfL, GFAP	0.823 (0.805 to 0.840)	0.040 (0.028 to 0.052)^a^	0.863 (0.839 to 0.887)	0.039 (0.024 to 0.055)^a^	0.796 (0.771 to 0.820)	0.040 (0.022 to 0.057)^a^
Model 3: Model 2+*APOE*4	0.808 (0.788 to 0.829)	Ref	0.854 (0.825 to 0.882)	Ref	0.781 (0.754 to 0.809)	Ref
M3 + AB42/40 ratio	0.814 (0.795 to 0.834)	0.006 (0.001 to 0.01)^a^	0.856 (0.828 to 0.883)	0.002 (−0.002 to 0.006)	0.79 (0.763 to 0.816)	0.008 (0.001 to 0.016)^a^
M3 + p-tau217	0.826 (0.807 to 0.845)	0.018 (0.009 to 0.026)^a^	0.872 (0.846 to 0.898)	0.018 (0.005 to 0.031)^a^	0.796 (0.769 to 0.823)	0.015 (0.003 to 0.027)^a^
M3 + NfL	0.816 (0.797 to 0.836)	0.008 (0.002 to 0.014)^a^	0.866 (0.839 to 0.892)	0.012 (0.001 to 0.023)^a^	0.786 (0.758 to 0.813)	0.004 (−0.001 to 0.010)
M3 + GFAP	0.819 (0.800 to 0.839)	0.011 (0.005 to 0.017)^a^	0.861 (0.834 to 0.888)	0.007 (0.001 to 0.014)^a^	0.795 (0.768 to 0.821)	0.014 (0.004 to 0.023)^a^
M3 + Aβ42/40, p-tau217, NfL, GFAP	0.835 (0.816 to 0.853)	0.026 (0.016 to 0.037)^a^	0.881 (0.857 to 0.906)	0.028 (0.012 to 0.043)^a^	0.805 (0.778 to 0.832)	0.024 (0.008 to 0.039)^a^

Abbreviations: Aβ42/40 = the ratio of amyloid-β 42 to amyloid-β 40; BMI = body mass index; GFAP = glial fibrillary acidic protein; MMSE = Mini-Mental Status Examination; NfL = neurofilament light; p-tau217 = phosphorylated tau protein 217.

Model 1 includes age, age,^2^ sex, ethnicity, education, smoking status, physical activity, BMI, hypertension, kidney function, type 2 diabetes, and cardiovascular disease.

The N cases/total in these models vary due to incomplete *APOE* genotype data; in Models 1 and 2, it is 448/5,225 and 348/4,281 in Model 3.

a Denotes 2-tailed difference at *p* ≤ 0.05.

## Discussion

In a large community-based longitudinal study, we found lower concentration of Aβ42/40 and higher concentration of NfL, p-tau217, and GFAP in plasma to be associated with higher risk of incident dementia over a follow-up of 13 years in survival analyses adjusted for numerous covariates. The C-index of these biomarkers ranged from 0.587 to 0.693 when considered individually and 0.760 when biomarkers were entered together in the prediction model. The specificity and NPV associated with the biomarkers were good, but sensitivity and PPV were modest to poor. The second approach to prediction modeling, that involved adding biomarkers to a model containing known risk factors of dementia, showed small, statistically significant increases in prediction with the addition of biomarkers: a maximum of 0.047 for all biomarkers together.

Studies based on data from research or memory-clinic settings, where biomarkers are tested against PET or CSF biomarkers for AD, have reported remarkably higher predictive values than those found in our study: AUCs of 0.90 and above for p-tau217,^5,8-10^ above 0.80 for Aβ42/40,^6,8^ and 0.70 for GFAP.^9^ These studies use unadjusted prediction models without other predictors, similar to the results presented in Figure 2. One cross-sectional study on 392 individuals with amyloid PET positivity as the outcome and the same biomarker panel as ours reported unadjusted AUC of 0.885 for p-tau217, 0.734 for Aβ42/40, 0.760 for NfL, and 0.756 for GFAP.^10^ Adding age, sex, and *APOE* genotype in that study led to values of 0.903, 0.786, 0.769, and 0.815, respectively.^10^ There are several explanations for the considerably poorer prediction in our study. One, our outcome was incident all-cause dementia rather than CSF or PET amyloid positivity, but this might not be a limitation as amyloid positivity is almost impossible to measure in general population settings. Furthermore, a recent study used data from 6 community-based autopsy studies (N = 4,354 decedents) to show high co-occurrence of AD and vascular pathologies.^33^ Two, the AUCs in research or memory-clinic settings are based on cross-sectional analyses, and it is possible that longitudinal prediction, with “onset” of amyloid positive status as the outcome, would result in lower AUCs than that reported in cross-sectional studies. Our results show better prediction at the short-term rather than long-term follow-up; for example, C-index for p-tau217 was 0.731 in the short-term and 0.662 in the long-term follow-up. Three, studies from research or memory-clinic settings are smaller and often have strict inclusion criteria. Our analyses are based on a large heterogeneous population as we wanted to examine the prognostic ability of blood-based biomarkers in real-world settings, an important condition for them to be considered as first-line screening tools.^34^

CSF and PET biomarkers are not affected substantively by chronic conditions, but this appears not to be the case for blood proteins.^24,35^ A number of common health-related conditions, sex, and *APOE* genotype are believed to modify concentrations of blood proteins.^36^ Furthermore, blood-based biomarkers are more strongly associated with brain Aβ levels in those without diabetes and cardiovascular conditions.^9^ Studies with strict inclusion criteria, that exclude individuals with certain chronic diseases, may show blood-based biomarkers to have better prediction than studies based on community-dwelling persons who have a range of chronic conditions. The role of *APOE* ε4 also seems to differ because it increases the AUC of plasma Aβ42/40-based prediction models with amyloid positivity as the outcome by about 0.10^22,23^ but much lower for all-cause dementia in community-based studies such as ARIC^19^ and in our study.

Recent population-based studies have primarily examined whether blood-based biomarkers have an association with either clinical AD or dementia in survival analyses adjusted for a range of covariates in follow-up ranging from 3 to 16 years^13^ These studies reported lower Aβ42^13^ and Aβ42/40,^17,19,20^ as well as higher p‐tau217,^16,21^ NfL,^13,15,16,18,20,21^ and GFAP^14,18,20,21^ to be associated with higher risk of all-cause or AD dementia. Three of these studies undertook prediction analyses; the first, based on the UK Biobank study (N = 48,582, 2.7% incident dementia cases, mean follow-up 13.2 years) with measures only of NfL and GFAP, reported an age alone model to have a C-index of 0.805 that increased to 0.871 on addition of these biomarkers and other covariates.^18^ The second study (ARIC, N = 1,523, 16.5% incident dementia cases, median follow-up 7.4 years) reported AUC of demographics alone to be 0.76 and that of biomarkers alone (Aβ42/40, p-tau181, NfL, and GFAP) to be 0.74, with the combined AUC being 0.81.^19^ The third study (Swedish SNAC-K, N = 2,148, 16.9% incident dementia cases, follow-up 10 years) chose to use cutoffs for biomarkers optimized for their study population and reported an AUC of 0.838 for p-tau217, NfL, and GFAP considered together.^21^

Besides the modest C-index, sensitivity, reflecting the ability to identify a true positive, was poor for all biomarkers in our study. Specificity, reflecting the ability to detect true negatives, was modest to good. For blood-based biomarkers in population settings, specificity may be more important than sensitivity as the next stage of investigations is invasive, costly, and has limited availability. The NPV for all tests was high, whereas PPV was low, as also previously shown in a prospective population-based study.^21^ Given the importance of prevalence in calculation of PPV and NPV, the use of these metrics in general population settings is moderately useful where prevalence is likely to be high only in the oldest age groups.

We add to the existing literature by explicitly considering 2 approaches to prediction for a better assessment of the potential utility of blood-based biomarkers for early detection of dementia in community-dwelling older adults. The first, by starting out with biomarker alone models, as in most studies that use amyloid positivity as the outcome. Besides modest C-index and poor sensitivity, there was also some evidence of better prediction in the short term rather than the long term with this approach. The second approach, consisting of testing whether blood-based biomarkers improve a model that includes readily available covariates, does not show substantial improvement in prediction. Taken together, our results suggest that these biomarkers may be useful in population settings where data on individuals' characteristics are limited, and biomarker levels can be used to select individuals for further investigation in specialized settings—a first-step screening tool due to high specificity of biomarkers.

The 2024 Alzheimer's Association criteria for AD include plasma p-tau217 as an independent diagnostic biomarker^37^; although we examined the predictive rather than diagnostic ability of biomarkers, it is possible that prediction would be better in individuals who already have cognitive symptoms with onset of clinical dementia imminent. The value of blood-based biomarkers was also questioned in a recent clinical cohort of individuals with subjective cognitive complaints or mild cognitive impairment that showed blood biomarker concentrations to be mainly explained by age, with minor contributions from cognitive function, measures of brain atrophy, and a genetic risk score, as opposed to CSF measures of these biomarkers.^35^

The diagnostic accuracy of the biomarker assays used in our study (ALZpath and Neuro 4-PlexE) is believed to be robust, with the ALZpath p-tau217 assay repeatedly ranked among the top-performing assays to detect AD pathophysiology.^38,39^ Although the impact of biomarker storage was not tested in our study, a recent study reported quantification of serum biomarkers not to be affected by the time they had been stored in freezers at −70°/−80°C.^40^ Of the 4 biomarkers considered in our study, p-tau217 has been shown to have the highest discrimination in previous studies^7,9,10,22-25,41^ and is most closely associated with both Aβ plaques and tau tangles^22^ irrespective of the specific assay used to measure biomarkers.^10^ It is believed to be a specific marker of AD-brain pathology rather than tauopathies such as frontotemporal dementia.^24^ A blood-based biomarker panel is believed to have superior predictive capability^6,42^ than any individual biomarker. Our results support both the findings on p-tau217 and the superior predictive ability of all biomarkers considered together (Figure 2). The precise cutoffs for blood-based biomarkers remain unclear, some studies have used cut points that maximized Youden index,^21^ others have used 1.5 SD,^43^ and several studies have used quartiles.^13,16,18,20,44^ We opted to use quartiles and provide the unstandardized values used for the cutoffs in the quartiles to allow comparison in future studies.

Key strengths of this study include the large, heterogeneous, population-based sample with a 13-year follow-up. There was virtually no loss to follow-up in the dementia ascertainment because of the use of electronic health records and linkage for all participants rather than ascertainment only on those who continue to participate in the clinical examination of the study. Dementia diagnosis was undertaken by physicians employed by the NHS in the United Kingdom, independently from the study investigators. The mean age at dementia diagnosis at around 80 years in our study matches that in the UK general population.^45^ The availability of a large range of covariates in the study allowed us to consider the role of sociodemographic, behavioral, and health-related factors in the analyses. A further advantage of the study is the use of a panel of biomarkers. The limitations of the study include incomplete data on dementia etiology, not allowing analyses on dementia subtypes. Participants in this study where mostly White, reflecting the UK population, and whether these findings apply to diverse settings is unclear. The use of electronic health records for dementia ascertainment also implies that milder cases of dementia may be missing, but whether this affects prediction findings is unknown.

In conclusion, in this study, based on 5,225 participants followed over 13 years, we found lower Aβ42/40 ratio and higher levels of p-tau217, NfL, and GFAP to be associated with a higher risk of all-cause dementia in analyses adjusted for a range of covariates. The predictive performance of these biomarkers on their own was modest and comparable with that for known risk factors that include sociodemographic, behavioral, and health-related factors. These findings suggest that blood-based biomarkers have limited utility for early detection of dementia in general population settings.

## Glossary

A β40: amyloid-beta 40
AD: Alzheimer disease
ARIC: atherosclerosis risk in community
AUC: area under the curve
Aβ42: amyloid-beta 42
BMI: body mass index
eGFR: estimated Glomerular Filtration Rate
GFAP: glial fibrillary acidic protein
HES: hospital episode statistics
MMSE: Mini-Mental Status Examination
NfL: neurofilament light
NHS: national health service
NPV: negative predive value
PPV: positive predictive value
SNAC-K: Study on Aging and Care in Kungsholmen
